# The Pathophysiology of Anorexia Nervosa in Hypothalamic Endocrine Function and Bone Metabolism

**DOI:** 10.7759/cureus.20548

**Published:** 2021-12-20

**Authors:** Keji Jada, Sandrine Kakieu Djossi, Anwar Khedr, Bandana Neupane, Ekaterina Proskuriakova, Jihan A Mostafa

**Affiliations:** 1 Research, California Institute of Behavioral Neurosciences & Psychology, Fairfield, USA; 2 Internal Medicine, California Institute of Behavioral Neurosciences & Psychology, Fairfield, USA; 3 Psychiatry, California Institute of Behavioral Neurosciences & Psychology, Fairfield, USA

**Keywords:** leptin and gherlin, hypotalamic gonadal axis, hypotalamic thyroid axis, hypotalamic adrenal axis, hypotalamic endocrine function, amenorrhea, bone metabolism, endocrinopathies, anorexia nervosa

## Abstract

Anorexia nervosa (AN) is a persistent psychiatric disorder that is marked by abnormal reduced weight and amenorrhea, which may be primary or secondary. AN affects multiple endocrine axes such as gonadal, thyroid, and adrenal axis, growth hormone, and insulin-like growth factor-1, adipokines such as leptin, gut peptides like ghrelin, peptide YY, and amylin. As a result of these changes bone mineral density is reduced, which increases the risk of bone fracture in patients. In this review, we focus on substantial endocrine alterations in AN with a particular emphasis on the severe bone loss associated with this condition and current bone therapies. The disorder primarily affects girls and women, who are the focus of this review. Although the majority of AN-related endocrinopathies improve over time, long-term consequences such as short stature, osteoporosis, and infertility may occur. To avoid serious consequences, nutrition therapy in these patients requires a full understanding of bone complications, and new therapeutic options for treatment should be researched.

## Introduction and background

Anorexia nervosa (AN) is a psychiatric condition characterized by an eating disorder with endocrine dysfunction. It mainly affects females during adolescence and its etiology is not yet clear or known. The prevalence of this condition among adolescents is around 1% with an annual incidence of new cases 10/100,000 between the ages of 15 and 19. The main characteristics of anorexia are underfeeding and fear of being overweight [1]. Patients attempt to maintain a below-normal weight by several means like vomiting after a meal, following a strict and unrealistic diet, use of diuretics, laxatives or enemas, starvation, and/or strenuous exercise. AN affects bones and the pituitary glands negatively due to starvation and malnutrition [2].

**Figure 1:**
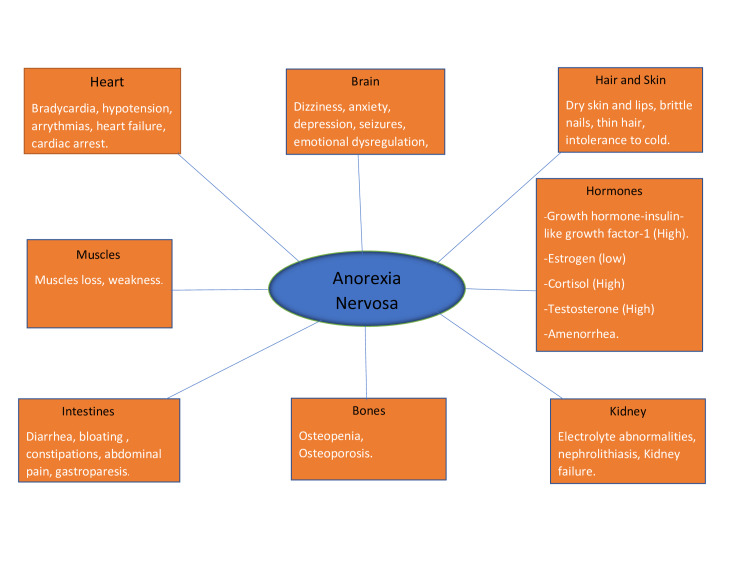
Effect of anorexia nervosa in multiple organs

Also, several hormonal changes occur including somatotropin resistance with low insulin-like growth factor-1 (IGF-1) levels, hypothalamic hypogonadism, relative hypercortisolemia, and changes in appetite-regulating hormones including leptin, ghrelin, and peptide YY (PYY). These contribute to abnormalities in bone metabolism resulting in low bone mass, impaired bone microarchitecture, and increased risk for fracture. They also negatively impact cognition, emotions, and mood. As AN affects women predominantly, amenorrhea is one of its serious complications [3]. We have summarized potential symptoms and complications of AN in multiple organs in Figure 1.

Physiological estrogen replacement should be used to improve bone resorption rates and measures of trait anxiety in adolescents with AN. The best strategy is to boost all biological outcomes such as a menstrual recovery and an electrolyte repletion for profound malnutrition and dehydration for weight gain. Other medications examined to improve bone results include testosterone and IGF-1 replacement, dehydroepiandrosterone (DHEA) with oral estrogen-progesterone combination tablets, bisphosphonates and teriparatide [3].

Although AN was first described centuries ago, the associated risk factors and etiology remain poorly understood and there is little data about it [4]. This article aims to debate the hormonal and bone disturbances that occur due to AN and possible therapeutic approaches for better outcomes and methods for low bone mineral density (BMD).

## Review

Hypothalamic-pituitary-gonadal axis

The most well-known endocrine complication of AN is amenorrhea which can be primary or secondary. Hypothalamic amenorrhea is believed to be caused by decreased energy availability in anorexic patients. In previous years, amenorrhea was a significant diagnostic criterion for AN, but because vulnerability to neuroendocrine dysregulation is exceedingly varied, the DSM-5 criteria no longer include amenorrhea as a crucial factor of the diagnosis acknowledging that many women with all the psychiatric features of anorexia and extremely low weight have regular menstrual cycles [1].

In 1974 Boyar et al. published an article in which he described multiple dysregulated luteinizing hormone (LH) pulsatility patterns in amenorrheic women with AN. The patient's LH pulsatile secretion resembled prepubertal or early pubertal girls with either exceptionally low pulse amplitude or increased during sleep. Studies at that time focused on the role of leptin as a regulator of gonadotrophin-releasing hormone pulsatility, which was thought to be responsible for amenorrhea in women with AN [5].

Some studies focused on kisspeptin (an endogenous ligand for the hypothalamic kisspeptin receptor) and its inactivating mutations that lead to pubertal failure. Kisspeptin has been observed to exhibit gonadotropin release in women with hypothalamic amenorrhea but there has been no research specifically examining its effect on AN [6].

Although weight loss usually precedes amenorrhea, some patients experience amenorrhea before weight loss and others reach an extremely low weight while still having a reproductive function [7]. This variation is likely due to individual variances in psychologic stressors, physical activity, fat mass, and leptin levels, resulting in a wide range of threshold weights required for ovulatory function [8,9]. Hypothalamic amenorrhea causes hypoandrogenemia and hypoestrogenemia in women with AN with a further decline in free testosterone levels in women who use oral contraceptives. These conclusions were discovered when a group of women with AN was compared to healthy controls [10]. It made sense because the ovaries secrete roughly 60% of testosterone in women of reproductive age whereas the adrenal precursors produced 40% [11]. Furthermore, investigations in adults found no decrease in the adrenal-derived prohormone dehydroepiandrosterone sulfate (DHEAS) compared to lean controls even though DHEA levels are generally stimulated by cosyntropin [10].

In contrast, compared to a laboratory-generated normal range, DHEAS levels in adolescent girls were shown to be low. If any, the effect of a relative androgen deficiency in women with AN remains unknown. Nonetheless, they may result in muscle or bone mass loss and adverse mood effects [12].

According to some studies, androgen levels have an inverse association with the degree of depression and anxiety symptoms in women with AN regardless of weight suggesting that relative androgen deficit may contribute to the severity of mood-related symptoms in these individuals. Low-dose testosterone in replacement dosages has been shown to have favorable mood effects in different adult female populations with few adverse effects. Thus, low-dose androgen treatment alone or in conjunction with other medicines may be a beneficial treatment strategy for managing mood comorbidities in women with AN but more research is needed [13].

The growth hormone (GH) insulin-like growth factor-1 (IGF-1) axis

In AN, high GH and low IGF-I levels indicate a nutritionally mediated acquired resistance to GH. Low amounts of GH-binding protein have also been reported. Refeeding can help to reverse abnormalities in the GH-IGF-I axis [14,15].

According to a deconvolutional analysis of GH pulse investigations in adolescents and adults, raised basal secretory rates and higher frequency of secretory bursts underpin the GH excess in AN [15]. These studies discovered a negative relationship between GH secretion and nutritional status markers as body mass index (BMI), fat mass, and leptin. Because acute fasting inhibits the liver's synthesis of IGF-I, GH excess in AN can be related in part to a lack of IGF-I-mediated negative feedback due to low IGF-I levels.

Hypothalamic abnormalities leading to excessive GH due to low somatostatinergic tone or high GH-releasing hormone have also been reported [16]. Muscle atrophy, growth failure, and bone loss are all possible consequences of GH resistance. Anabolic effects of GH on bone are direct and indirect (through IGF-I). The only medication that has been demonstrated to improve BMD in women with AN is recombinant human IGF-I; however, it is not yet permitted for this use [17].

Hypothalamic-pituitary-thyroid axis

In people with AN, hypothyroidism symptoms such as bradycardia, hypothermia, hypotension, dry skin, and a slowed metabolic rate are prevalent. Due to defective peripheral conversion, anorexic patients have a similar biochemical constellation of thyroid hormone abnormalities to a sick euthyroid syndrome with low triiodothyronine (T3) levels and low to normal thyroxine (T4) levels. Thyroid-stimulating hormone levels are usually normal to slightly below average in AN patients [18]. These patients are not considered hypothyroid despite evidence of peripheral thyroid hormone shortage as evidenced by a delayed Achilles reflex half-relaxation time and subsequent improvements with exogenous T3 [19].

Thyroid hormone levels are altered for a variety of reasons in people with AN. In anorexia, like in any chronic disease or starvation, peripheral deiodination of T4 to T3 is reduced and conversion to inactive reverse T3 is enhanced [20]. The hypothalamic release of thyrotropin-releasing hormone may be suppressed in AN resulting in a weak thyroid-stimulating hormone response to low peripheral thyroid hormone levels [19]. A normal or delayed response in thyroid-stimulating hormone can be caused by exogenous thyrotropin-releasing hormone and this delay reverses with weight gain [21]. Malnutrition followed by low IGF-1 levels is most likely the cause of thyroid atrophy in AN. Thyroid atrophy may increase depressive feelings and continued hunger in anorexic patients who have a smaller overall thyroid volume than age-matched control participants. [22].

As previously stated, the biochemical thyroid abnormalities were seen in AN usually resolve with weight gain. But unfortunately, gaining weight by itself will not alleviate psychological issues or personality attributes. Thyroid disease is not the primary cause of psychological disorder although it can exacerbate pre-existing psychological difficulties [23].

Hypothalamic-pituitary-adrenal axis

In AN, plasma cortisol levels rise regardless of whether urinary-free cortisol levels are elevated or normal [24]. According to Weiner in his study, there is evidence that the low T3 syndrome has been shown to lengthen cortisol's half-life in plasma by delaying its metabolic clearance. Also, the HPA appears to be activated as well. The cortisol production rate is considerably raised in AN and the number of secretory episodes of cortisol is increased when calculated for body mass and body surface area [25]. As a result, when dexamethasone is given to anorexic patients, their plasma cortisol levels are partially suppressed like that observed in patients with depression and Cushing's disease. HPA activation is unknown; however, increased synthesis of corticotropin-releasing hormone (CRH) or hypersensitivity of adrenal cortical cells to CRH stimulation is thought to be among the possibilities [26].

Recently Foppiani et al. demonstrated that desmopressin does not stimulate adrenocorticotropic hormone (ACTH) and cortisol in AN patients. This could be linked to the downregulation of hypophyseal 1-deamino-8D-arginine vasopressin (DDAVP) V3 receptors in AN [27]. The dexamethasone test has no medical significance in acute AN conditions however, it may have predictive value in individuals who are regaining weight [28]. Refeeding studies of anorexic patients have indicated that weight gains of as little as 10% are related to the normalization of cortisol levels regardless of the initial weight [27].

 Adipokines and appetite-regulating hormones

In AN, adipokines like adiponectin and leptin (an anorexigenic hormone) are disrupted as are other appetite-regulating hormones including ghrelin (orexigenic) and PYY. Because of reduced basal leptin secretion and pulse amplitude, people with AN have low leptin levels. Adequate leptin concentrations allow the hypothalamic-pituitary-gonadal axis to function normally and reduced leptin levels in AN may lead to inadequate gonadotropin production [29]. Women with AN with higher fat mass and leptin levels are more likely to have menstruation than women with AN and similar weight despite their low weight. Systemic leptin promotes bone growth especially in appendicular and cortical bone whereas central leptin affects axial trabecular bone due to its effects on the sympathetic nervous system. Decreased leptin levels have been linked to lower bone formation indicators and BMD in healthy populations and AN patients. Compared to a normal-weight population, adiponectin levels are higher, constant, or lower in AN. These variations could be due to various assays identifying distinct circulating adiponectin isoforms as the proportion of lower-molecular-weight adiponectin to total levels in AN is higher than in controls [30].

Ghrelin promotes the production of GH and ACTH while decreasing the production of LH and follicle-stimulating hormone. In adults and adolescents with AN, increased basal secretion and secretory pulse amplitude boost ghrelin levels. Higher ghrelin levels are a suitable adaptation to drive food intake during hunger, produce more elevated GH and cortisol release, and decrease gonadotropin secretion. Ghrelin may also have anabolic effects on the bones. Increased ghrelin levels predict higher BMD in healthy adolescents but not in AN suggesting ghrelin resistance in AN [29].

Although some studies in adults find unchanged or reduced levels of PYY (an anorexigenic hormone), PYY levels are higher in adults and adolescents with AN compared to controls. Higher PYY levels are associated with lower fat mass. PYY can influence gonadotropin secretion and is detrimental to osteoblast function. Higher PYY levels are linked to lower bone turnover markers in adolescents with AN and lower BMD in adults [31].

Central peptides changes in anorexia nervosa

It has been suggested but not definitively proven that peptide changes, even when caused by malnutrition or abnormal eating habits, may have a role in the origin and maintenance of some clinical elements of AN altering the course and prognosis. Tortorella et al. focused on the physiology of central and peripheral peptides that significantly impact eating behavior, body weight, and energy homeostasis in AN patients [32]. In Table 1, we highlight some of the changes in central peptides that regulate eating behavior and energy homeostasis in people with AN [33-41].

**Table 1:** Summary of some of the central peptides changes in AN AN: Anorexia nervosa; CSF: Cerebrospinal fluid; ND: No difference from healthy controls; Increased: Higher than healthy controls; Decreased: Lower than healthy controls; AGRP: Agouti-related peptide; BDNF: Brain-derived neurotrophic factor; CRH: Corticotropin-releasing hormone; TRH: Thyrotropin-releasing hormone; SRIF: Somatostatin; α-MSH: α-melanocyte-stimulating hormone; NPY: Neuropeptide Y

	Changes in AN		Reference
	Acute Phase (AN)	Weight restored (AN)	
Plasma AGRP	Increased	No Difference	Merle et al. (2011) [33]
Serum BDNF Plasma BDNF	Decreased Increased	Decreased	Brandys et al. (2011) [34]
CSF CRH	Increased	Increased	Hotta et al. (1986) [35]
Plasma Oxytocin	Decreased	ND	Lawson et al. (2012) [36]
CSF TRH	Decreased	Decreased	Lesem et al. (1994) [37]
CSF SRIF Plasma SRIF	Decreased Increased	ND	Kaye et al. (1988) [38]
Plasma α-MSH	ND	ND	Moriya et al. [39]
CSF NPY Plasma NPY	Increased ND	Increased ND	Sedlackova et al. (2011) [40]
CSF Vasopressin Plasma Vasopressin	ND/Decreased ND/Decreased	ND/Decreased ND/Decreased	Gold et al. (1983) [41]

Bone consequences in anorexia nervosa

Osteopenia is a common and typically long-term complication of AN resulting in clinical fractures and an increased risk of fracture throughout life. A study of women with AN in their mid-twenties found that 55% of them had osteopenia and 35% had osteoporosis, with only 15% having a normal BMD at all skeletal locations studied. In people with AN, the annual rate of loss in BMD at the spine and hip is about 2.5% [42].

A study by Vestergaard et al. described an increase in fracture risk. Reduced markers of bone production and higher markers of resorption have been observed in adults leading to the concept that osteopenia is caused by low osteoblast and high osteoclast activity. On the other hand, AN causes a generalized drop in bone turnover markers during adolescence, which is generally a time of substantial bone turnover [43].

During adolescence, the impact of AN on bone is especially concerning. Adolescence is a period of increasing bone formation as a person approaches peak bone mass, which is a crucial indicator of future bone health and fracture risk. Bone accrual rates are substantially reduced in adolescents with AN resulting in a steady fall in bone density Z-scores [44]. In addition, considerable changes in body weight and composition, pubertal growth, and pubertal hormones such as estradiol and IGF-I occur in anorexic patients impacting bone metabolism [45].

AN affects both cortical and trabecular bone; however, trabecular bone is more severely impacted than cortical possibly reflecting the substantial impact of estrogen deprivation [46]. Dual-energy x-ray absorptiometry (DXA) observations of reduced bone state are corroborated by evaluating bone microarchitecture using ultrahigh-resolution peripheral quantitative computed tomography. The volume and thickness of bone trabecular are reduced in girls but trabecular separation increases. Anorexic patients may experience abnormalities in bone microarchitecture before experiencing changes in BMD [47].

Hypogonadism and later menarchal age are significant contributions to low BMD associated with AN. Estrogen reduces the activity of the receptor activator of nuclear factor k-B ligand (RANKL) and limits the release of proinflammatory cytokines through increasing osteoprotegerin (OPG). Proinflammatory cytokines are prevalent in AN, which may contribute to bone loss. OPG levels are high in AN and are inversely related to BMD despite low estrogen levels [48]. Reduced sclerostin, a product of the *SOST* gene and a glycoprotein released by osteocytes, has also been associated with the effects of estrogen on bone. Sclerostin reduces osteoblast activity by blocking Wnt signaling and increases osteoclasts by boosting RANKL activation of the receptor activator of nuclear factor k-B [49]. Higher sclerostin levels are related to increased bone turnover indicators in controls but this association is interrupted in AN. In anorexic females, physiologic estrogen replacement improves BMD without affecting sclerostin levels [50]. Other hormones that are affected by AN are displayed in Table 2 [30].

**Table 2:** Bone anabolic hormones affected by anorexia nervosa BMD: Bone mineral density; ↓: Decreased

	Levels in Anorexia	Association with bone
Insulin	↓	Low BMD
Amylin	↓	Low BMD
Oxytocin	↓	Low BMD

Current treatments for bone disease

Various therapeutic techniques have been investigated for the treatment of bone disease in AN with variable outcomes. In hypogonadal women, estrogen replacement with oral contraceptives makes intuitive sense but there is no proof that exogenous estrogen is genuinely effective.

Although bisphosphonates improve bone density, maintaining a healthy body weight has a more significant long-term impact on bone density. The use of bisphosphonates in young women with AN presents obvious issues as these women may wish to become pregnant once they have recovered and the safety of these medications during pregnancy has yet to be proven. Studies of bisphosphonates have been observed to exhibit improper calcium homeostasis and fetal bone growth in pregnant rats [51].

Menatetrenone (vitamin K2) has shown promise to reduce vertebral bone loss, boost bone formation markers, and decrease bone resorption signs in patients with AN [52]. In those with AN, DHEA levels are abnormally low, which is likely linked to bone loss. Exogenous DHEA use does not significantly increase bone density even though it may improve psychological aspects [12].

Bone development is promoted by recombinant IGF-1, which inhibits bone resorption. It improves bone health in people with GH insufficiency (by stimulating bone metabolism and converting vitamin D to 1,25-dihydroxycholecalciferol) [53]. Recombinant IGF-1 therapy may benefit patients with AN who have a functional GH deficit due to GH resistance. Combining the anabolic capabilities of recombinant IGF-1 with antiresorptive abilities of oral contraceptives has been demonstrated to improve bone density [17].

Nutritional rehabilitation and behavioral support for bodyweight maintenance are essential and should be the cornerstones of AN treatment. Unfortunately, adolescent bone deficiency can lead to a lifelong loss in bone density even if weight is regained [54].

Limitations

There were several limiting factors while we searched for materials for this review. Our data came primarily from open-access articles written in English. Hence, some closed-access articles and articles published in other languages may have been missed.

## Conclusions

AN is a primary psychiatric disorder characterized by severe endocrine disorders and severe bone loss. Nutritional rehabilitation usually reverses abnormalities in the adrenal and thyroid glands; nevertheless, there may be long-term consequences such as short stature, infertility, and osteoporosis. Some problems persist after recovery and may contribute to illness progression and relapse. Currently, there are no FDA-approved medications for low BMD treatment in anorexia nervosa; therefore, weight gain and menstruation recovery remain the most effective strategy to increase BMD. In adolescent AN, physiologic estrogen replacement (mostly transdermal) improves BMD and studies are ongoing to assess the influence of IGF-1 replacement on bone density and structure. Further research is essential to better understand the disease's etiology and complications and to find effective treatments for AN and its comorbidities.
